# Cellular environment of TTR deposits in an animal model of ATTR—Cardiomyopathy

**DOI:** 10.3389/fmolb.2023.1144049

**Published:** 2023-03-08

**Authors:** Cristina Teixeira, Helena Sofia Martins, Maria João Saraiva

**Affiliations:** ^1^ i3S—Instituto de Investigação e Inovação em Saúde, University of Porto, Porto, Portugal; ^2^ IBMC—Instituto de Biologia Molecular e Celular, University of Porto, Porto, Portugal

**Keywords:** transthyretin, amyloidosis, cardiomyopathy, extracellular matrix, macrophages

## Abstract

**Introduction:** Cardiac amyloidoses are the most fatal manifestation of systemic amyloidoses. It is believed the number of cases to be greatly underestimated mostly due to misdiagnosis. Particularly, the involvement of TTR V30M in the heart of ATTRV30M amyloidosis has not been completely understood specifically in terms of implicated cellular pathways, heart function and cardiac physiology. In the present work we proposed to characterize TTR V30M cardiac involvement particularly at the tissue cellular level in a mouse model.

**Methods:** HSF ± hTTR V30M mice, a model that expresses human TTRV30M in a Ttr null background, widely used for the characterization and modulation of neurological features of ATTRV30M amyloidosis was used. SDS-PAGE of cardiac homogenates followed by Western blot was performed. Immunohistochemistry and double immunofluorescence analyses were carried out to determine TTR deposition pattern and sub-localization.

**Results:** Western blots were able to detect TTR in its monomeric state at ∼14 kDa. Immunofluorescent images showed TTR was found mostly in the intercellular spaces. Blood contamination was excluded by CD31 staining. Tissues were Congo Red negative. Upon TTR and macrophages (CD68) staining in the cardiac tissue a clear tendency of macrophage convergence to the tissue regions where TTR was more abundant was observed. Moreover, in some instances it was possible to detect co-localization of both fluorophores. Cardiac fibroblasts were stained with PDGFr-alpha, and here the co-localization was not so evident although there was some degree of co-occurrence. The hearts of transgenic mice revealed higher content of Galectin-3.

**Conclusion:** This animal model and associated features observed as result of cardiac TTR deposition provide a promising and invaluable research tool for a better understanding of the implicated pathways that lead to the lethality associated to TTR cardiac amyloidosis. New therapeutic strategies can be tested and ultimately this will lead to improved treatment alternatives capable of increasing patient’s quality of life and life expectancy and, hopefully to eradicate a condition that is silently spreading worldwide.

## Introduction

ATTRV30M amyloidosis remains the most well studied type of hereditary amyloidosis and while the majority of symptoms are neuropathic, up to 43% of patients also exhibit cardiac amyloidosis (Gertz et al., 2015). To this date, ATTRV30M amyloidosis patients with severe cardiac involvement still have very limited cardiac medical supportive treatment with the standard drugs used for the treatment of heart failure being inefficient or unsafe in this particular context. The pathways and cellular mechanisms involved in the cardiac pathology need further investigation.

The cardiac extracellular matrix (ECM) is a dynamic structure composed by different proteins that form a three-dimensional network essential not only for structural support but also for signalling transduction, while functioning as a reservoir for growth factors and proteases that can be activated following injury (Weber, 1989; Kong et al., 2014; Valiente-Alandi et al., 2016).

Excessive ECM dynamics is a feature of ATTRV30M amyloidosis with ECM-related genes having been reported as upregulated in salivary glands of ATTRV30M amyloidosis patients. MMP9, particularly, was found upregulated in tissues with amyloid aggregates (Sousa et al., 2005). Histochemical analyses of cardiac tissue also revealed higher levels of laminin, fibronectin, collagen IV and heparan sulfate which were positively correlated to TTR amyloid fibrils in the basement membrane of cardiomyocytes (Misumi et al., 2009).

Cardiac fibroblasts, the cells responsible for ECM homeostasis, are the main sources of collagen synthesis and, at the same time, producers of metalloproteinases (MMPs), the leading enzymes that degrade ECM. After a cardiac insult and during the reparative process, activated macrophages secrete galectin-3 which is able to induce fibroblast proliferation and collagen deposition (Sharma et al., 2004).

To further assist in the understanding of ATTR V30M effect in the cardiac ECM structure and dynamics, we aimed to study a particular animal model, HSF ± hTTR V30M transgenic mice (Santos et al., 2010) which has been extensively investigated in the context of peripheral and central nervous systems in ATTRV30M amyloidosis and it has proved crucial for the understanding of the pathogenesis of ATTR amyloidogenesis as well as in the preclinical evaluation of pharmacotherapy and gene therapy studies (Cardoso et al., 2010; Ferreira et al., 2014; Butler et al., 2016; Ferreira et al., 2016). In the present work we focus particularly on the presence of macrophages and fibroblasts in the heart.

## Materials and methods

### Animal procedures

Transgenic mice expressing the human TTR V30M in a Ttr null background, heterozygous for the heat shock transcription factor 1 (Hsf1) (named HSF ± hTTR V30M) and mice expressing endogenous Ttr, heterozygous for Hsf1 (named HSF ± mTTR wt) were used in our studies (*n* = 6/group unless stated otherwise). Both mouse strains are in the 129/Sv background. Ages ranged between 6 and 20 months.

All animals were maintained at 24°C ± 1°C, humidity 45%–65%, under a 12 h light/dark cycle and fed with regular chow and tap water *ad libitum*. Euthanasia was performed with an overdose of anaesthetics containing ketamine (75 mg/kg) and medetomidine (1 mg/kg) by intraperitoneal injection. Blood was collected and mice were perfused with a phosphate buffered saline (PBS) solution. Hearts fragments were frozen at −80°C, fixed in 4% neutral buffered formalin and embedded in paraffin; or conserved in Optimal cutting temperature compound (OCT compound) for cryosectioning. All animal experiments were approved by the Portuguese General Veterinarian Board (authorization number 014982 from DGV-Portugal) and animals were kept and used strictly in accordance with National rules and the European Communities Council Directive (86/609/EEC), for the care and handling of laboratory animals.

### Immunohistochemistry (IHC)

Immunohistochemistry was performed in 3 μm-thick heart sections that were deparaffinised in xylene (Fisher Scientific), and hydrated in descendent ethanol series. For antibodies raised in mice, we used a Mouse on Mouse (M.O.M) immunodetection kit (Vector Labs) following the manufacturer’s instructions. Endogenous peroxidase activity was inhibited with 3% hydrogen peroxide in PBS for 30 min. When necessary, antigen unmasking was performed with a sodium citrate solution at 98°C for 15 min.

Sections were blocked in blocking buffer (1% BSA and 10% FBS in PBS) for 1 h at room temperature. Primary antibodies were added for an overnight incubation at 4°C: rabbit polyclonal anti-TTR (1:200, Dako), for GAG (glycosoaminoglycan) detection, mouse monoclonal anti-heparan sulfate (1:50, Amsbio), anti-chondroitin sulfate (1:50, Abcam). Secondary antibodies were biotinylated anti-rabbit (1:200, Vector Labs) or biotinylated anti-mouse provided in the M.O.M kit according to the datasheet. Antigen visualization was achieved using Vectastain Elite ABC Reagent (Vector Labs) and for colour development we used 3,3′-diaminobenzidine (Sigma–Aldrich) as substrate. Tissue sections were examined with an Olympus BX50 light microscope equipped with an Olympus DP71 digital camera for image acquisition.

### Immunofluorescence

Immunofluorescence was performed in 3 μm-thick heart sections that were deparaffinised in xylene (Fisher Scientific), and hydrated in descendent ethanol series. When necessary, antigen unmasking was performed with a sodium citrate solution at 98°C for 15 min or with a 20 μg/mL proteinase K solution for 20 min at room temperature. Immunofluorescence was also carried out in 8 μm-thick frozen sections.

Both paraffin-embedded and cryopreserved sections were blocked in blocking buffer (1% BSA and 10% FBS in PBS) for 1 h at room temperature. Primary antibodies were added for an overnight incubation at 4°C: rabbit polyclonal anti-TTR (1:100, Dako), mouse monoclonal anti-α-sarcomeric actin (1:500, Sigma-Aldrich), goat polyclonal anti-CD31 (1:50, Santa Cruz), rabbit polyclonal anti-collagen IV (1:50, Abcam), rat monoclonal anti-CD68 (1:50 Abcam), and goat polyclonal anti-PDGFr-α (1:200, R&D systems). Secondary antibodies were Alexa Fluor-488 anti-rabbit, Alexa Fluor-568 anti-mouse, Alexa Fluor-594 anti-rat, Alexa Fluor-594 anti-goat all at 1:1,000, from Molecular Probes. Double staining was achieved by simultaneous antibody incubation or sequential incubations. Autofluorescence was quenched with a 0.3% Sudan Black in 70% ethanol solution for 10 min. Coverslips were mounted in Vectashield with 4,6-diamidino-2-phenylindole (DAPI, Vector Labs) and observed under a Laser Scanning Confocal Microscope Leica TCS SP5 II.

### Congo red staining

The detection of amyloid in cardiac tissue sections was attempted after Congo red staining and visualisation under polarised light (Puchtler and Sweat, 1965). Briefly, 5 µm-thick sections were deparaffinised in Histoclear (National Diagnostics), hydrated in descendent ethanol series, and incubated with 0.01% NaOH in 80% ethanol saturated with NaCl. Sections were then stained with a 0.5% Congo red (Sigma-Aldrich) solution in 80% ethanol saturated with NaCl, counterstained with hematoxylin and mounted with Entelan (Merck).

Slides were observed in an Olympus BX50 light microscope equipped with a DP71 digital camera using a polariser.

### Western blotting

Mice heart fragments from HSF ± mTTR wt (*n* = 6) and HSF ± hTTR V30M (*n* = 6) were homogenised with a glass rod homogeniser in Radioimmune Precipitation Assay buffer (Santa Cruz) supplemented with PMSF, protease inhibitor cocktail and sodium orthovanadate provided with the kit. The supernatant from the centrifuged lysates was collected and the protein concentration was determined using the Bio-Rad assay kit (Bio-Rad). 50 μg protein or 1 µL of plasma sample were then mixed with 4x Laemmli buffer containing 240 mM Tris-HCl, 8% SDS, 40% glycerol, 0.04% bromophenol blue and 10% 2-mercaptoethanol and incubated for 5 min at 95°C. An additional well was loaded with 100 ng recombinant TTR to aid in the identification of specific TTR bands in animal samples. Proteins were separated by SDS-PAGE and transferred to 0.2 µm nitrocellulose membranes (GE Healthcare Bioscience). For TTR visualization, blocking was performed overnight at 4°C with 5% nonfat dry milk in TBS plus 0.1% Tween 20. Membranes were incubated with rabbit polyclonal anti-TTR (1:300, Dako) for 2 h at room temperature. Otherwise, membranes were blocked with 5% nonfat dry milk in TBS plus 0.1% Tween 20 for 1 h at room temperature following incubation with anti-periostin (1:1,000 Abcam), anti-galectin-3 (1:2,000 Cedarlane) or anti-GAPDH (1:100.000, Abcam).

Detection was achieved using horseradish peroxidase-conjugate secondary antibody anti-rabbit (1:5,000, The Binding Site), anti-mouse (1:5,000, Thermo), and visualized with Clarity Western ECL (Bio-Rad). Immunoblot quantitative analysis was performed using the Image Lab software (Bio-Rad).

### Statistics

Statistical analysis was performed using GraphPad Prism 6. Cell culture data are presented as the mean ± SEM and depict the average of at least three independent experiments.

Ratio-paired *t*-test was used to compare cell blots. Mann-Whitney statistics were applied in the remaining semi-quantitative analyses. Whenever *p* values were less than 0.05 the differences were considered statistically significant (*p < 0.05; **p < 0.01; ***p < 0.001).

## Results

### Evidence of human TTR V30M in the murine heart

**FIGURE 1:**
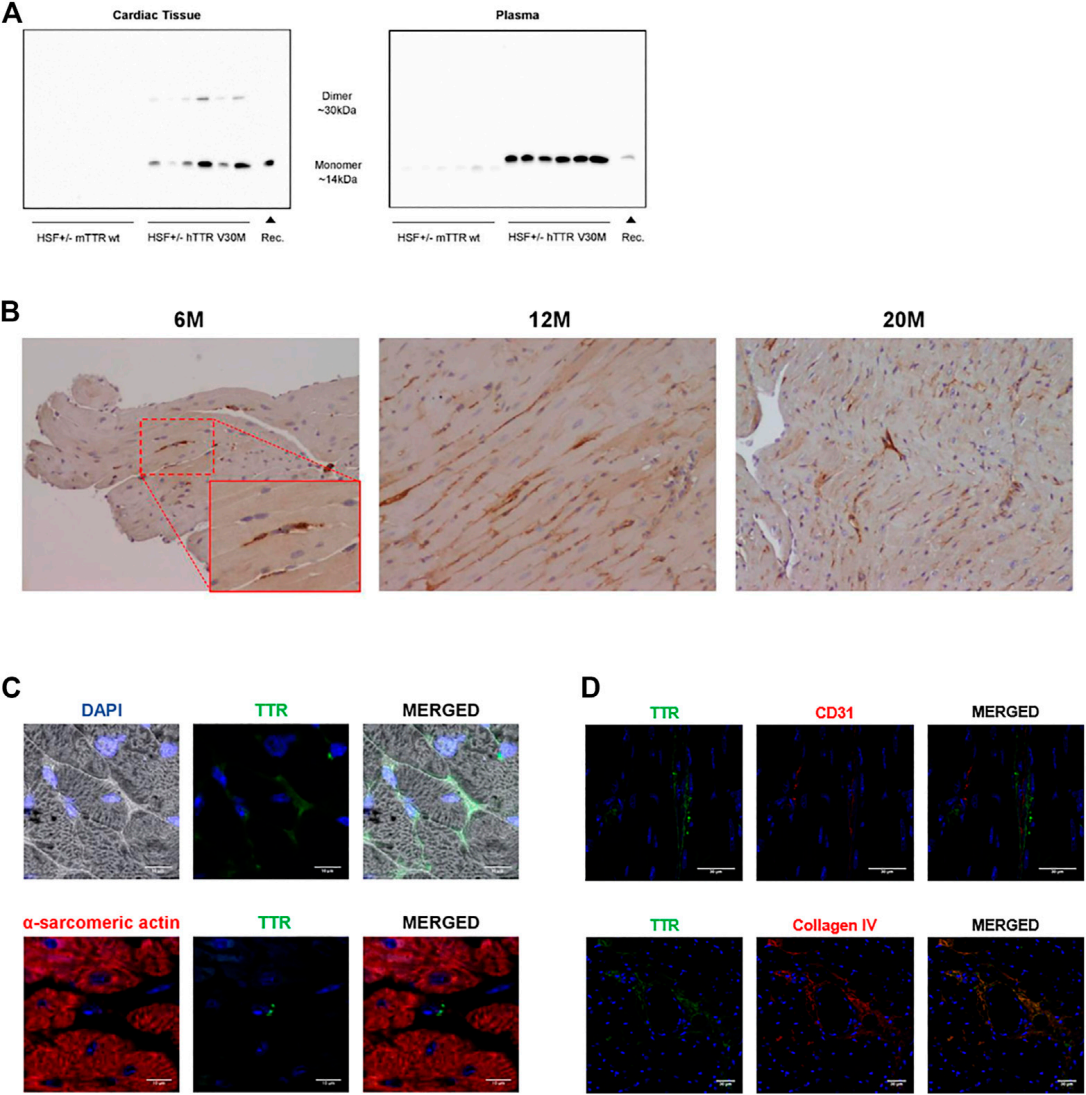
**(A)** TTR detection in cardiac homogenates and plasma samples. Immunoblot analysis of TTR levels in the cardiac tissue and plasma samples of HSF ± mTTR wt (*n* = 6) and HSF ± hTTR V30M (*n* = 6). TTR monomer was detected in both tissue and plasma samples whereas the dimeric form of this protein only appeared in tissue. **(B)** TTR deposition pattern in the cardiac tissue. Immunohistochemistry staining TTR in paraffin-embedded cardiac sections from HSF ± hTTR V30M mice at 6, 12, and 20 months. Magnification 20x**. (C)** TTR subcellular localization in the cardiac tissue. Confocal microscopy of paraffin-embedded heart sections of HSF ± hTTR V30M mice. In the upper row a differential interference contrast (DIC) channel was added to facilitate visualization of tissue structure. On the lower row, alpha-sarcomeric actin for cardiomyocyte staining appears red. TTR is stained green and nuclei are stained blue with DAPI. Scale bars, 10 µm. **(D)** TTR detection outside blood vessels. Confocal microscopy of paraffin-embedded heart sections of HSF ± hTTR V30M mice. **(A)** TTR staining is present on the outside of blood vessels, here stained with CD31. **(B)** Image depicting a large blood vessel with TTR co-localizing with collagen IV in the basement membrane. Nuclei are stained blue with DAPI. Scale bars, 30 µm.

It is well established that HSF ± hTTR V30M mice have TTR deposition in the gastrointestinal tract, skin and peripheral and autonomic nervous system but TTR deposition in the cardiac tissue had not yet been evaluated in detail. By performing SDS-PAGE of cardiac homogenates followed by Western blot we were able to detect TTR in its monomeric state at ∼14 kDa and as a dimer ∼30 kDa with a stronger band in the former (left panel of Figure 1A). An additional well was loaded with 100 ng recombinant TTR to ascertain the specificity of the bands from the whole cardiac tissue. Human TTR was also detected in its monomeric conformation in plasma samples of HSF ± hTTR V30M (right panel of Figure 1A).

Histological analyses were then carried out to determine TTR deposition pattern and sub-localization. Immunohistochemistry revealed TTR in the cardiac tissue in animals from a young age of 6-months and throughout life, i.e., at 12 and at 20 months of age (Figure 1B). The staining pattern remained similar with aging with no apparent increased TTR levels in the older animals.

To better understand subcellular localization, we resorted to immunofluorescence microscopy of cardiac sections. Immunofluorescent images showed that TTR was found mostly in the intercellular spaces (Figure 1C). We also stained cardiomyocytes, the cell type constituting most of the cardiac volume, and major contributors to cardiac dysfunction. The purpose was to evaluate if there was any intracellular TTR that may be exerting a direct effect in cell viability or function. The observed double-staining of cardiomyocytes (with alpha-sarcomeric actin) and TTR (with an anti-TTR antibody) clearly showed that TTR was not inside these cells. Moreover, we were able to observe small TTR aggregates in close proximity to non-cardiomyocyte nuclei, which lead us to speculate that besides the extracellular matrix, TTR was probably occurring in other cardiac cell types (Figure 1C lower row).

### Observed cardiac TTR is not from blood contamination

The heart is a highly irrigated organ with a vast network of vessels so we wanted to exclude the hypothesis of the observed TTR being due to blood contamination despite the fact that our animals were perfused with a saline solution prior to sacrifice and organ harvest. Thus, we aimed to stain blood vessels, more specifically the endothelium, the inner cell lining of blood vessels with one of the most common markers, CD31. By double staining with CD31 and TTR, we would be able to distinguish between circulating TTR, if present, or TTR deposited in the tissue. Our results indicate that the observed TTR is not inside blood vessels (Figure 1D) although it is still possible to find residual amounts associated with blood. We also proceeded to stain collagen IV, one of the main components of the basement membrane that is often a deposition site for TTR. Indeed, we observed co-localization of TTR and collagen IV, corroborating the previous result indicating that the majority of TTR deposition occurs outside the vasculature, in the extracellular matrix (Figure 1D lower row).

### No cardiac amyloid deposition nor evidence of amyloid-associated proteins

Positive Congo red staining remains the gold standard in the confirmation of amyloid deposition in tissue samples. Therefore, we scanned cardiac slides looking for evidence of the typical pinkish coloration found in amyloid deposits in tissues upon Congo red staining. The results were very clear in showing no detection of amyloid aggregates in any of the cardiac structures even in 20-months old animals (data not shown). We went further and looked for proteins commonly associated with amyloid to evaluate if they were over-represented despite the absence of amyloid deposits. Glycosaminoglycans (GAGs) are found associated with amyloid deposits but are also known to accelerate the aggregation of different amyloidogenic proteins. More specifically, a research work has demonstrated the ability of both heparan sulfate and chondroitin sulfate (the latter with lower efficiency) to accelerate TTR aggregation (Bourgault et al., 2011; Noborn et al., 2011). Being two of the most common GAGs, we also looked at the staining patterns of these sulfated GAGs in the cardiac tissue of these mice. Similarly, for both cases, there was no staining that proved the presence of either of the molecules in the hearts of HSF ± hTTR V30M mice in any particular region (data not shown).

### Matricellular proteins

**FIGURE 2:**
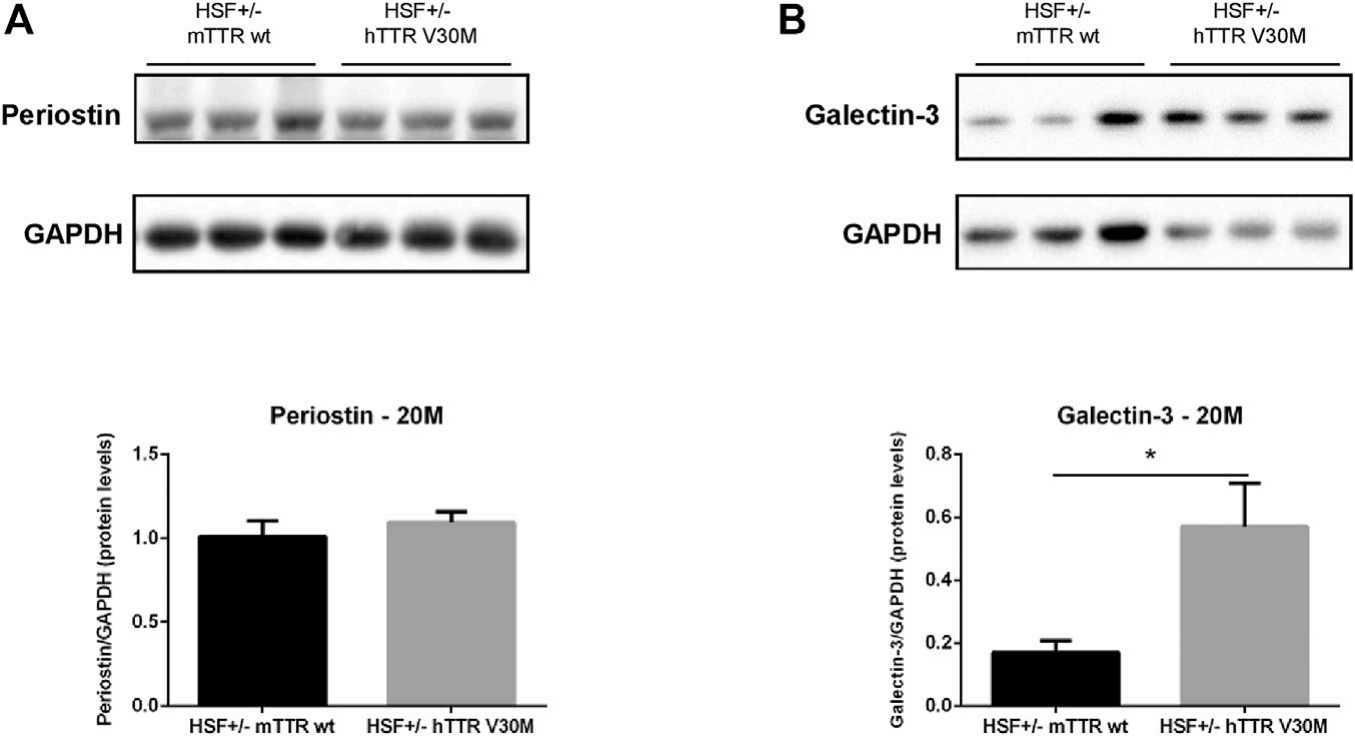
Protein levels of relevant matricellular molecules. Representative immunoblot analyses of Periostin **(A)**, and Galectin-3 **(B)**. Qualitative results are shown below. *n* = 6 for each animal group. Error bars, S.E.M. *p < 0.05.

Matricellular proteins are secreted molecules that are not usually part of the normal ECM and do not play a significant role in its structure, but do participate in the regulation of cellular signalling. Our studies focused on periostin, a unique and major contributor of ECM remodelling in the heart; and galectin-3, an established biomarker of heart failure that binds ECM components and transduces or modulates fibrogenic signalling cascades. Periostin cardiac protein levels were semi-quantified but no differences were detected between both animal groups (Figure 2A). The hearts of HSF ± hTTR V30M, however, revealed prominently higher (>300%) galectin-3 protein content than the control group (Figure 2B).

### Intracellular TTR–Macrophages and fibroblasts

**FIGURE 3:**
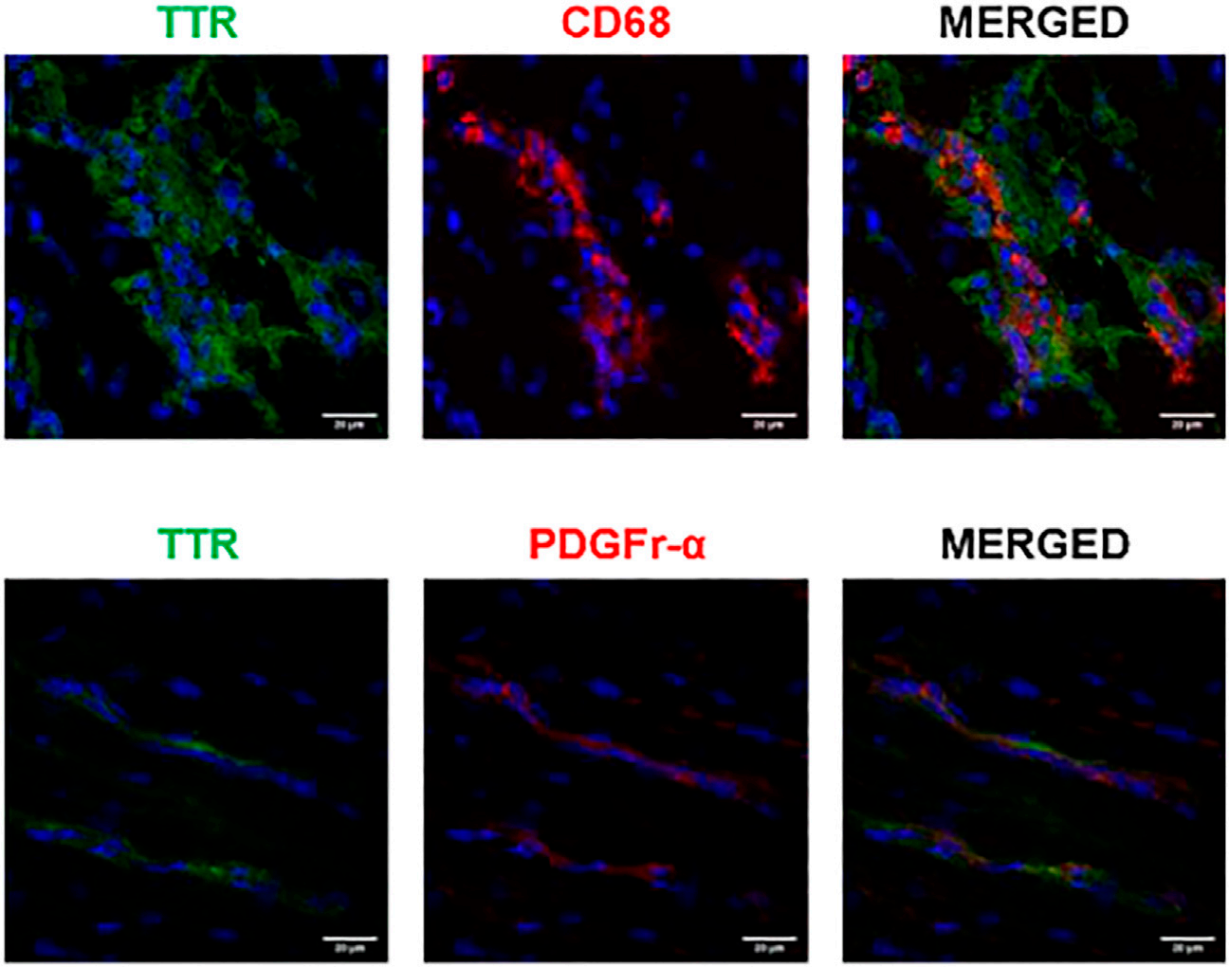
Possible TTR uptake by macrophages. Confocal microscopy of frozen heart sections of HSF ± hTTR V30M mice. Macrophage (CD68) presence was more prevalent in TTR deposited sites whereas there is no clear internalization of TTR by cardiac fibroblasts, here stained with PDGFr-alpha. Nuclei are stained blue with DAPI. Scale bars, 20 µm.

Both macrophages and fibroblasts have been described to co-localize with TTR in the skin and gastrointestinal tract of TTR V30M mice (Misumi et al., 2013). The same authors demonstrated co-localization between fibroblasts and TTR in ATTR V30M patients, both in the skin and cardiac tissue. Our results upon TTR and macrophages (CD68) staining in the cardiac tissue of HSF ± hTTR V30M mice, showed a clear tendency of macrophage convergence to the tissue regions where TTR was more abundant. Moreover, in some instances it was possible to observe co-localization of both fluorophores (Figure 3, upper row). Cardiac fibroblasts were stained with PDGFr-alpha, and here the co-localization was not so evident although there was some degree of co-occurrence (Figure 3, lower row).

## Discussion

In the present work we wanted to further understand the cardiac implications of TTR V30M in ATTR amyloidosis. By studying HSF ± hTTR V30M, a mice model widely used for the characterization and modulation of neurological features of ATTRV30M amyloidosis, we wanted to determine the existence of similarities found in this animal model and ATTR V30M amyloidosis patients in terms of the presence of amyloid aggregates and pattern of TTR deposition.

The amount of TTR found in the cardiac tissue was quite significant from an early age of 6 months. We paid particular attention to the left ventricle when analysing the distribution of TTR deposition. Left ventricle is the most common site for TTR deposition in human patients and it is the cardiac compartment that suffers the most alterations leading to cardiac dysfunction. However, in our mice, we did not detect a preferential site for the deposition of TTR nor amyloid.

On a cellular level, it is well established that TTR deposition occurs mostly in the extracellular matrix which agrees with our observations. Still, while observing cardiomyocytes in heart sections stained for TTR, we were inclined to hypothesize that it also occurs in non-myocyte cells. In a previous study from our group, the analysis of cardiac biopsy samples from ATTR V30M patients revealed TTR inside a small number of cardiac fibroblasts and macrophages, showing the capacity of these cells to internalize small protein aggregates (Misumi et al., 2013). Cardiac fibroblasts play a very important role in the ECM homeostasis. Not only do they participate in the synthesis of ECM proteins but they are also responsible for the secretion of the most important enzymes that degrade ECM components, the metalloproteinases (MMPs). Likewise, macrophages, which constitute only 7%–8% of non-myocyte cells (Heidt et al., 2014; Pinto et al., 2016), play a pivotal role in ECM remodelling and also contribute to the clearance of small TTR aggregates. We stained cardiac fibroblasts with PDGFr-alpha, a receptor tyrosine kinase described as uniquely expressed by fibroblasts in the adult heart (Ali et al., 2014; Moore-Morris et al., 2014; Kanisicak et al., 2016; Pinto et al., 2016; Ivey et al., 2018). Our observations did not allow us to clearly state that these cells are uptaking TTR aggregates besides being in close proximity to them. Moreover, the observed reduced fibroblast immunoreactivity in the cardiac tissue, suggests a sub-representation of the cardiac fibroblast population, which is known to be very heterogeneous. It is quite possible that the studies previously mentioned (Misumi et al., 2013), that used a different fibroblast marker (S100A4), stained different populations of these cells, possibly more involved in TTR protein clearance. On the other hand, the “uniqueness” of PDGFr-alpha as a cardiac fibroblast marker has been challenged in different studies demonstrating the expression of this protein in cells that share characteristics with mesenchymal stem cells (Chong et al., 2011; Chong et al., 2013; Noseda et al., 2015; Kim et al., 2017).

In HSF ± hTTR V30M mice, the higher macrophage convergence to the TTR deposition sites prompt us to speculate that necessary signalling cascades are being activated in order to trigger a basal, although insufficient, clearance mechanism, most likely as an attempt to alleviate the TTR cardiac burden. The importance of macrophage activity has been highlighted in a previous study by Michalon et al., where it was reported that TTR aggregates removal involves activation of phagocytic immune cells including macrophages, mediated by specific antibodies (Michalon et al., 2021).

We were rather surprised to find no traces of amyloid aggregates even in animals that were reaching the end of life. It appears that the cardiac environment and intrinsic features in these animals do not propitiate the formation of amyloid fibrils. On the contrary, these animals present amyloid deposition in the GI, particularly in stomach.

The remarkable increased galectin-3 levels found in the cardiac tissue of HSF ± hTTR V30M was an interesting finding. Galectin-3 is predominantly expressed and secreted by activated macrophages (Frunza et al., 2016) and has been proposed as a biomarker for cardiac fibrosis (Lin et al., 2009; Lopez-Andres et al., 2012), described as being able to activate fibroblasts (MacKinnon et al., 2008; Frangogiannis, 2018) and suggested to act as a matricellular protein capable of transducing or modulating fibrogenic signalling cascades (Mackinnon et al., 2012). The higher amount of galectin-3 found in the HSF ± hTTR V30M myocardium can be a reflex of macrophage activation due to the presence of TTR aggregates. As demonstrated earlier, CD68^+^ macrophages were more likely to be found in cardiac regions where TTR aggregates were more prevalent. Interestingly, there is no scientific consensus regarding galectin-3 involvement in fibrosis. While some studies provide evidence of its vital role in fibrosis and dysfunction of the remodelling heart (Yu et al., 2013; Martinez-Martinez et al., 2015) other investigations using galectin-3 knockout mice demonstrated that this is not a critical modulator of cardiac fibrosis but may be able to delay the hypertrophic response (Frunza et al., 2016; Nguyen et al., 2018).

In summary, we present an animal model with demonstrated TTR deposition in the cardiac tissue that may be having a negative impact in the cardiac remodelling process. Deposited TTR in the cardiac ECM may be promoting proliferation and convergence of macrophages to the aggregates. Possibly, these macrophages are the main contributors to the enhanced galectin-3 protein levels detected in HSF ± TTR V30M mice.

This animal model and associated features observed as result of cardiac TTR deposition provide a promising and invaluable research tool for a better understanding of the implicated pathways that lead to the lethality associated to TTR cardiac amyloidosis. New therapeutic strategies can be tested and ultimately this will lead to improved treatment alternatives capable of increasing patient’s quality of life and life expectancy and, hopefully to eradicate a condition that is silently spreading worldwide.

## Data Availability

The raw data supporting the conclusion of this article will be made available by the authors, without undue reservation.
